# Fluorescent proteins reveal what trypanosomes get up to inside the tsetse fly

**DOI:** 10.1186/s13071-018-3204-y

**Published:** 2019-01-04

**Authors:** Wendy Gibson, Lori Peacock

**Affiliations:** 1 School of Biological Sciences, University of Bristol, Bristol, BS8 1TQ UK; 2 School of Clinical Veterinary Science, University of Bristol, Langford, Bristol, BS40 7DU UK

**Keywords:** *Glossina*, Tsetse, *Trypanosoma brucei*, Sexual reproduction, Meiosis, Gametes, Fluorescent proteins

## Abstract

The discovery and development of fluorescent proteins for the investigation of living cells and whole organisms has been a major advance in biomedical research. This approach was quickly exploited by parasitologists, particularly those studying single-celled protists. Here we describe some of our experiments to illustrate how fluorescent proteins have helped to reveal what trypanosomes get up to inside the tsetse fly. Fluorescent proteins turned the tsetse fly from a “black box” into a bright showcase to track trypanosome migration and development within the insect. Crosses of genetically modified red and green fluorescent trypanosomes produced yellow fluorescent hybrids and established the “when” and “where” of trypanosome sexual reproduction inside the fly. Fluorescent-tagging endogenous proteins enabled us to identify the meiotic division stage and gametes inside the salivary glands of the fly and thus elucidate the mechanism of sexual reproduction in trypanosomes. Without fluorescent proteins we would still be in the “dark ages” of understanding what trypanosomes get up to inside the tsetse fly.

## Background

Keith Vickerman’s iconic image of the life-cycle of *Trypanosoma brucei* in the mammalian and tsetse fly hosts [1] is well known to parasitologists - indeed, it is the cover illustration of J. D. Smyth’s parasitology textbook [2]. This very detailed diagram was the culmination of several decades of research using light and electron microscopy, at the time the key research tools available to investigate parasite life-cycles. In this review, we want to show how the use of fluorescent proteins, an experimental approach that could not have been foreseen in 1985, has helped move this story forward (and perhaps something to bear in mind when you hit an impasse in your own research: the techniques you need may not yet have been invented).

In 2008, Osamu Shimomura, Martin Chalfie and Roger Tsien were awarded the Nobel prize for Chemistry for their work on the discovery of green fluorescent protein, GFP, and its application to biological research. The efforts of these three scientists made it possible to make living, fluorescent cells and whole organisms, a tremendous boon to many researchers, parasitologists included. Approaches incorporating the use of GFP were quickly adopted by trypanosomatid researchers e.g. [3, 4], and in the context of our research, fluorescent proteins turned the tsetse fly from a “black box” into a bright showcase to track trypanosome migration, development and mating inside the insect. Here we describe some of our results to illustrate how fluorescent proteins have helped to elucidate what trypanosomes get up to inside the tsetse fly.

## Sexual reproduction

### Design of experimental crosses

An important omission from Vickerman’s diagram of the *T. brucei* life-cycle are stages involved in sexual reproduction; this is not surprising, as it was believed at the time that *T. brucei* reproduced asexually by binary fission. The first experimental evidence for genetic exchange in *T. brucei* appeared in 1986 when it was shown that hybrids were produced after co-transmission of two genetically distinct strains through the tsetse fly [5]. However, because the hybrids were found to have DNA contents higher than expected for a diploid, it was uncertain whether this was true sexual reproduction involving meiosis and haploid gametes, or some kind of fusion creating a polyploid hybrid with subsequent loss of genetic material to return to the diploid state [6, 7]. The precise mechanism remained elusive, because of the complexity of the developmental cycle of *T. brucei* in the tsetse fly and the small numbers of trypanosomes available for analysis. For genetic exchange, the life-cycle stages of interest are those found in the salivary glands of the fly [8, 9], and as these are difficult to culture *in vitro*, experiments on genetic exchange involve co-transmission of the parental trypanosome lines through tsetse flies. While many experimental flies develop a midgut infection, particularly if fed substances such as glutathione that suppress their antimicrobial immune responses [10], few go on to develop a salivary gland infection. This low success rate means it is rare to find flies with a co-infection, a prerequisite for finding hybrids. Hence, for several years research on the mechanism of genetic exchange in trypanosomes made little headway.

**Fig. 1:**
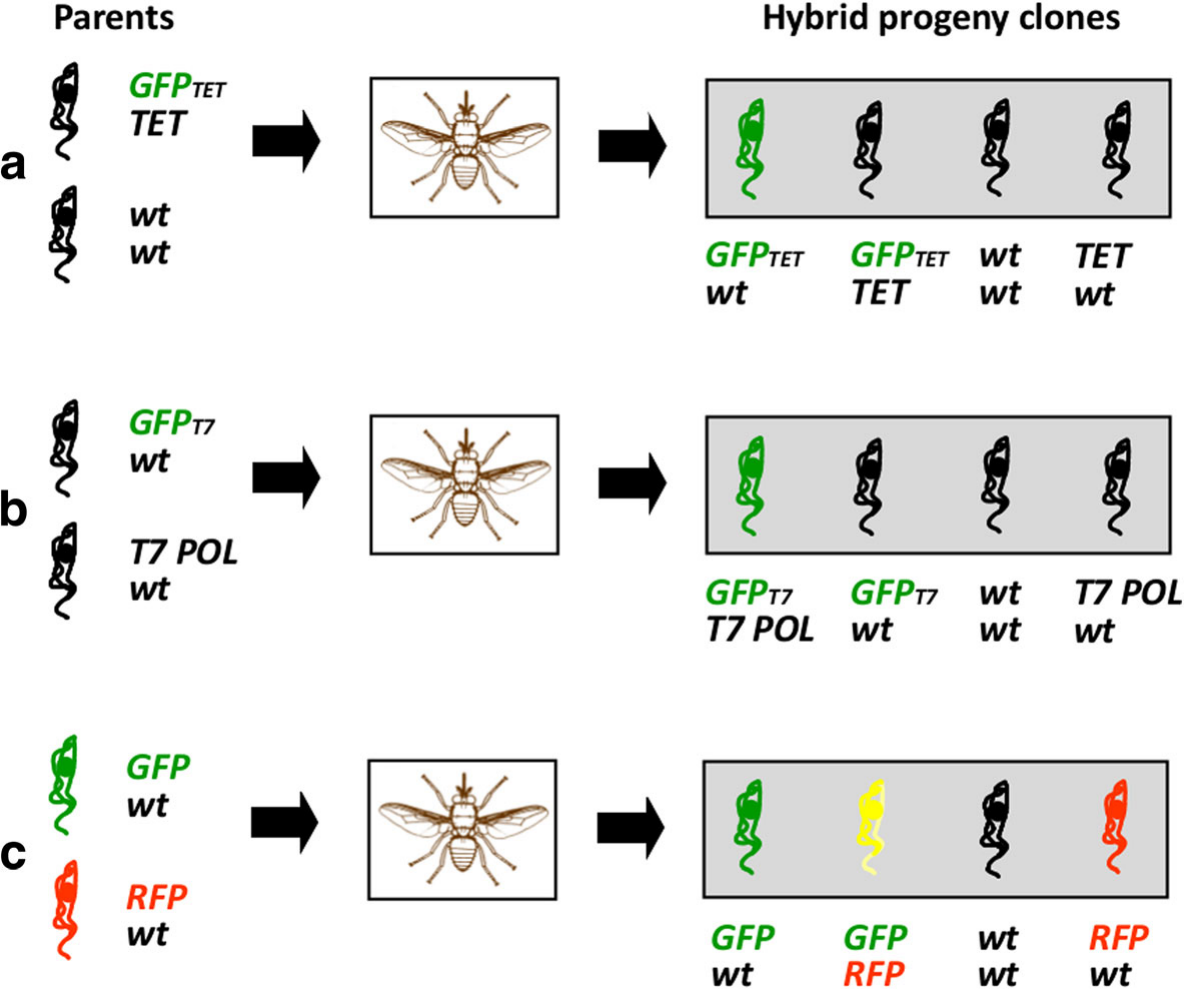
Design of experimental crosses. Sequential experimental designs (**a**-**c**) used to investigate mating in *Trypanosoma brucei*. Diploid parental trypanosomes were genetically engineered to contain the genes indicated: *GFP*, gene for green fluorescent protein; *GFP*_*TET*,_ *GFP* gene under control of the *TET* repressor; *TET*, gene for bacterial *TET* repressor; *GFP*_*T7*,_ *GFP* gene driven by phage T7 promotor; *T7 POL*, gene for phage T7 polymerase; *RFP*, gene for modified red fluorescent protein. Parental trypanosome clones were co-transmitted through tsetse flies and the expected genotypes of hybrid progeny, assuming Mendelian inheritance, are as indicated

Finding hybrids in experimental crosses had been largely a matter of luck, like finding a needle in a haystack [5, 11]. Hence a feature of the design of our first experimental cross using GFP was that hybrid, but not parental, trypanosomes should be fluorescent, as if hybrids could wave their hands and say “I’m here!”. Encouraged by the finding that even a single green fluorescent trypanosome could be detected inside a salivary gland by fluorescence microscopy, we set up an experimental cross using a recombinant trypanosome line expressing the Tet repressor, with a GFP gene under control of the Tet repressor on another chromosome, such that the GFP gene would be released from repression if the two chromosomes segregated independently during mating (Fig. 1a) [12]. The experimental design proved moderately successful in that green fluorescent hybrid clones were isolated from the salivary glands of one fly [12]; however, GFP expression also occurred spontaneously through loss of expression of the Tet repressor, so not all fluorescent cells were necessarily hybrids.

Our next approach was to cross recombinant lines where one parent contained GFP driven by the T7 promotor and the other parent supplied the T7 polymerase, so that only hybrids that received transgenes from both parents would fluoresce (Fig. 1b). Although hybrid clones were generated in these crosses, no fluorescent trypanosomes were produced, perhaps because expression from the strong T7 promotor fatally disrupted normal transcription in the ribosomal RNA locus.

The third experimental design was both simple and effective. Red and green fluorescent trypanosomes were crossed, such that a quarter of the progeny would inherit both RFP and GFP genes and appear yellow fluorescent (Fig. 1c) [13]. This experimental design had the major advantage that salivary glands with a mixed infection could easily be identified and taken forward for analysis, while those containing only a single parental trypanosome were discarded. This was a significant improvement in the efficiency of finding hybrids, as time was no longer wasted in futile analysis of single parental infections. In our first red/green cross, nearly every fly with one or both salivary glands containing a mixed infection of red and green fluorescent trypanosomes produced hybrids [13], leading to the conclusion that mating was not such a rare event as previously believed. We realised that the limiting factor for mating was whether both parental trypanosomes colonised the same salivary gland.

The red/green experimental design guaranteed success and we used it to investigate whether trypanosomes were capable of intraclonal mating as well as outcrossing by attempting to cross red and green fluorescent trypanosomes derived from the same clonal lineage [14]; we found that intraclonal mating was rare compared to outcrossing, suggesting that trypanosomes can distinguish self and non-self genotypes and might have mating types like other single-celled eukaryotes. This question remains unanswered, despite analysis of a large series of F1, F2 and back crosses, all based on the red/green cross design [15]. We also investigated whether the trait of human infectivity conferred by the serum resistance associated (*SRA*) gene [16, 17] was inherited by hybrid progeny, creating new genotypes of human-infective trypanosomes. This had been predicted by population genetics analysis of the microsatellite genotypes of a large collection of *T. brucei* isolates, which produced clear evidence of admixture between human infective (*T. b. rhodesiense*) carrying the *SRA* gene and non-human-infective (*T. b. brucei*) lacking the *SRA* gene [18]. Experimental crosses of three different strains of *T. b. rhodesiense* with various *T. b. brucei* strains yielded hybrid progeny, some of which had inherited the *SRA* gene and were resistant to lysis by human serum *in vitro* [19], confirming that new genotypes of *T. b. rhodesiense* can be produced by sexual reproduction between human infective and non-human-infective trypanosomes.

## Meiosis and gametes

**Fig. 2:**
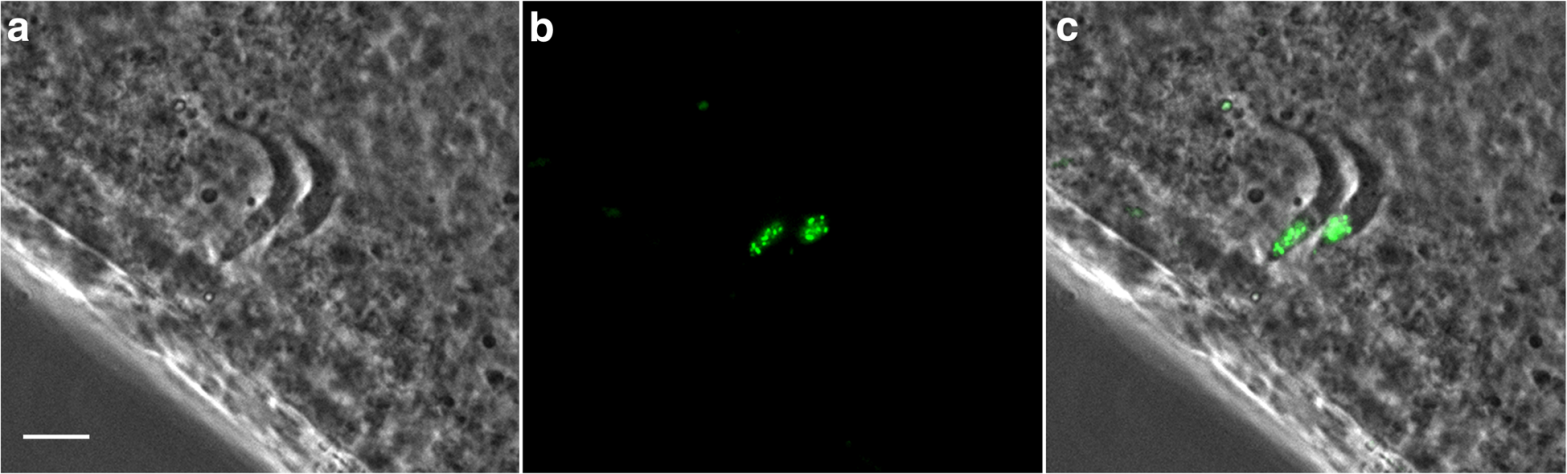
Meiotic dividers in the salivary gland. Live phase contrast and epifluorescence images of trypanosomes of *Trypanosoma brucei brucei* strain J10 expressing the fusion protein YFP::DMC1 inside a tsetse salivary gland. Trypanosomes expressing the fluorescent fusion protein have the nucleus very near the posterior end. **a** Phase contrast. **b** Fluorescence. **c** Merge. *Scale-bar*: 5 μm

Although genotype analysis of parental and progeny clones had produced convincing evidence that meiosis was involved in genetic exchange in *T. brucei* [20], direct demonstration of a meiotic division was lacking. Comparative analysis of genome sequence data revealed that *T. brucei* had genes for several of the key meiosis-specific proteins [21], opening the possibility of functional analysis. In collaboration with Mark Carrington, we tested whether expression of these meiosis-specific proteins could be detected during the trypanosome life-cycle by tagging them with yellow fluorescent protein, YFP. To our delight, we found that three meiosis-specific proteins, MND1, DMC1 and HOP1, were expressed in the nucleus of a small proportion of dividing epimastigote trypanosomes in the salivary glands, and nowhere else [22]. These meiotic dividers had a characteristic morphology with two kinetoplasts and flagella and a large, posterior nucleus (Fig. 2). Crossing one of the YFP-tagged lines with a red fluorescent trypanosome answered the question whether meiosis occurred before fusion or *vice versa*: only one instance of a hybrid trypanosome expressing both RFP and the tagged meiosis protein was detected, indicating that meiosis normally occurs before fusion [22].

**Fig. 3:**
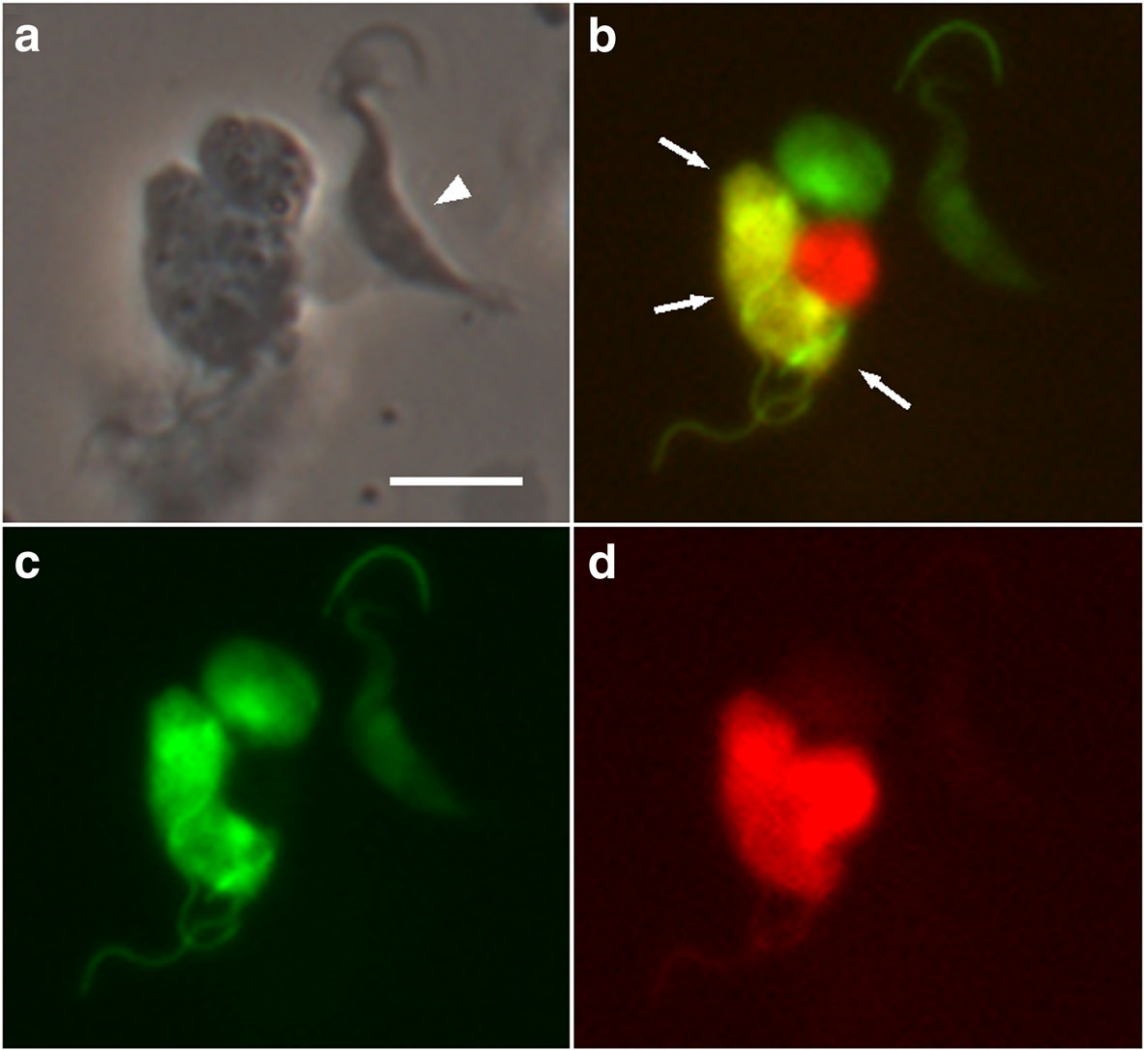
Interactions and cytoplasmic exchange between gametes. Red fluorescent (J10 RFP) and green fluorescent (1738 GFP) trypanosomes separately derived from tsetse salivary glands at day 20 post-infection and mixed *in vitro*. The cluster contains several gametes characterised by their small, pear-shaped bodies and relatively long flagella; the righthand trypanosome (arrowhead) is a dividing epimastigote. The cluster contains five trypanosomes, three of which show both red and green fluorescence (arrows), indicating that they have exchanged cytoplasm. **a** Phase contrast. **b** Red and green fluorescence. **c** Green fluorescence. **d** Red fluorescence. *Scale-bar*: 10 μm

The meiotic dividers appeared early on during colonisation of the salivary glands by migratory trypanosomes from the fly gut, suggesting that gametes should also be found in the salivary glands around this time point. Without genetic markers for gametes, we needed another way to identify them. Since we had already established that fusion occurred after meiosis, it seemed possible that gametes might be found in close association prior to fusion, and hence we mixed together salivary gland-derived trypanosomes of single parental origin *in vitro* and searched for interacting red and green trypanosomes (Fig. 3). We observed interacting pairs where the two cell bodies were held close together while the flagella of the two trypanosomes were intertwined; interacting pairs typically consisted of a particular type of cell with a short, pear-shaped body and relatively long flagellum [23]. Within the observation timeframe of about an hour, some red and green fluorescent trypanosomes had exchanged cytoplasm giving rise to yellow fluorescent cells (Fig. 3); it remains to be demonstrated that such cells have exchanged DNA as well as cytoplasm. Analysis of salivary gland-derived trypanosomes by cell morphology and DNA content demonstrated that the putative gametes had a haploid DNA content relative to metacyclics [23], confirming that they are the likely products of meiosis.

## Development of trypanosomes in tsetse

**Fig. 4:**
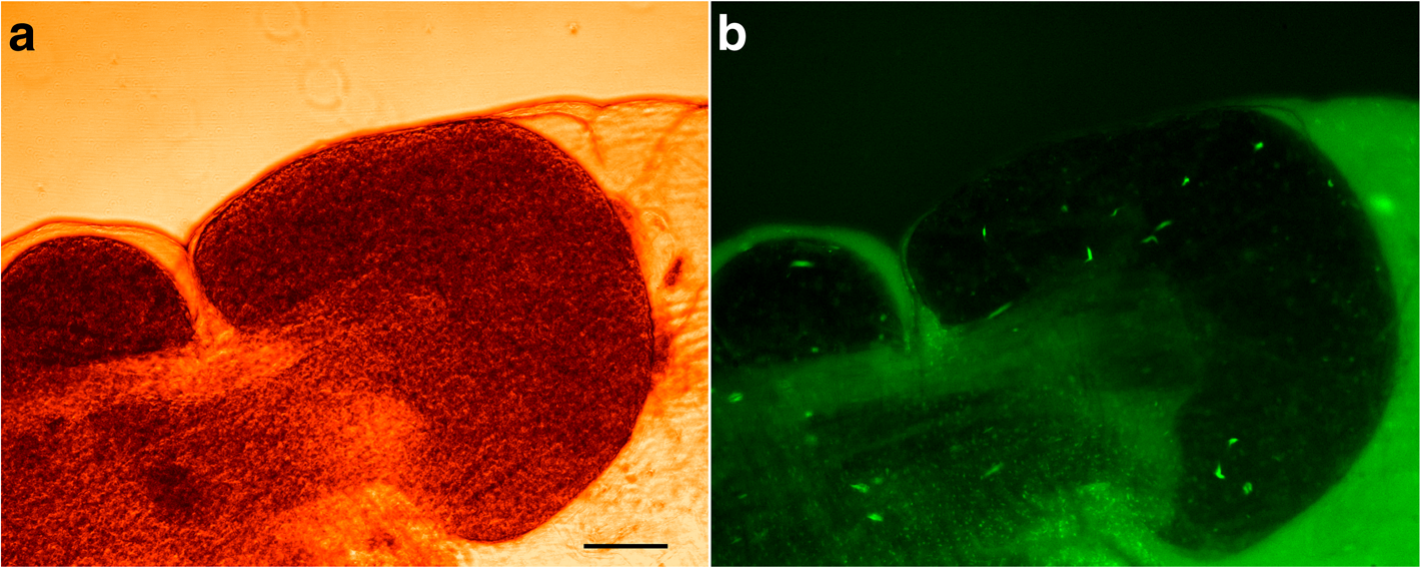
Fluorescent trypanosomes within the bloodmeal inside the midgut. Green fluorescent trypanosomes (*Trypanosoma brucei gambiense* strain TH2) visualised in the bloodmeal of a tsetse fly 48 hours after the infected bloodmeal. **a** Brightfield image showing the upper extent of the bloodmeal in the anterior midgut. *Scale-bar*: 100 μm. **b** Fluorescence image revealing small numbers of green fluorescent trypanosomes distributed throughout the bloodmeal. Copyright: Creative Commons Attribution 4.0 License (https://creativecommons.org/licenses/by/4.0/). Citation: Gibson & Bailey (2003) The development of *Trypanosoma brucei* in the tsetse fly midgut observed using green fluorescent trypanosomes. *Kinetoplastid Biology and Disease*. 2003;2:1 [24]

**Fig. 5:**
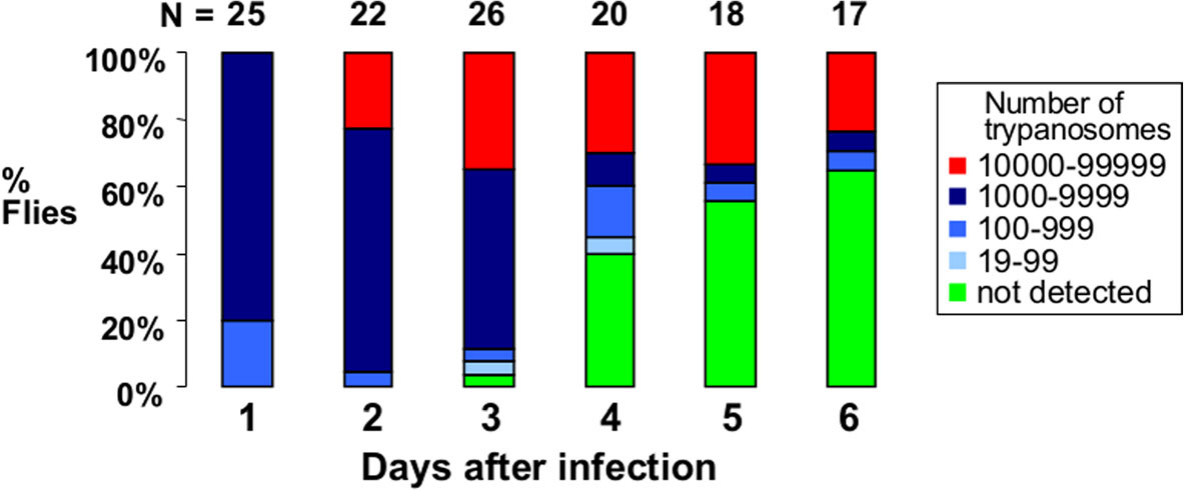
Attrition of trypanosome infection in midgut. Numbers of trypanosomes (*Trypanosoma brucei gambiense* strain TH2) present in individual tsetse flies (*Glossina morsitans*) on days 1–6 after infection. N = number of individual flies examined at each timepoint. Midgut infections have been divided into 5 categories according to the number of trypanosomes. Data from [24]

**Fig. 6:**
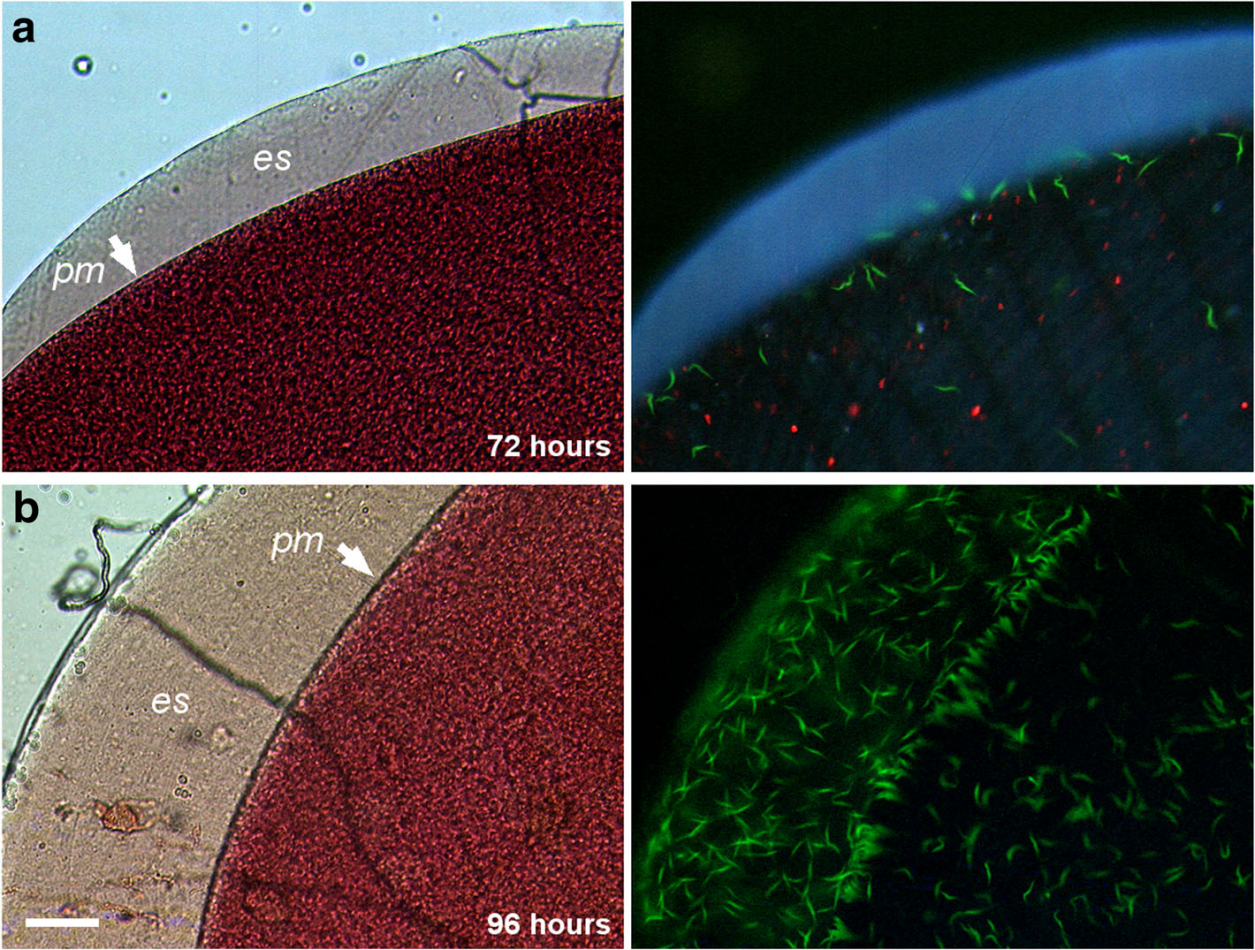
Invasion of the ectoperitrophic space. Green fluorescent trypanosomes (*Trypanosoma brucei gambiense* strain TH2) visualised in the bloodmeal of a tsetse fly 72 hours (**a**) and 96 hours (**b**) after the infected bloodmeal. Each panel shows the brightfield image (left) and fluorescence image (right). In **a** the bloodmeal is held within the peritrophic matrix (PM, arrowed) and trypanosomes are restricted to the endoperitrophic space. In **b** the trypanosomes have invaded the ectoperitrophic space (*es*). *Scale-bar*: 50 μm. Copyright (panel **b**): Creative Commons Attribution 4.0 License (https://creativecommons.org/licenses/by/4.0/). Citation: Gibson & Bailey (2003) The development of *Trypanosoma brucei* in the tsetse fly midgut observed using green fluorescent trypanosomes. *Kinetoplastid Biology and Disease*. 2003;2:1 [24]

Just as fluorescent trypanosomes have proved invaluable for elucidating the mechanism of sexual reproduction in trypanosomes, they have also provided a window into the developmental cycle of trypanosomes in tsetse. Green fluorescent trypanosomes were used to reveal the sequence of events from the moment the fly imbibed a bloodmeal containing bloodstream form trypanosomes [24]. In this cell line, GFP transcription was driven by the procyclin promotor, a strong Pol I promotor used by midgut procyclics to express their major surface proteins, procyclins [25, 26]. As the procyclin promotor is down-regulated in bloodstream form trypanosomes (BSF) [27], the BSF fed to the flies in the infective bloodmeal were not detectably fluorescent but became brightly fluorescent after 4 hours when they had transformed to procyclics; this enabled us to calculate that < 10% of BSF successfully transformed to procyclics. Despite this initial tenfold decrease in numbers, a thriving population of procyclics was found in the midguts of all infected flies, growing in numbers from days 1–3 after infection (Fig. 4). However, from day 3 onwards, a proportion of flies became negative, such that by day 5 over half the flies had eliminated their midgut infection (Fig. 5) [24]. This no doubt resulted from the action of the tsetse innate immune system, which harnesses powerful immune effector molecules, such as anti-microbial peptides, lectins and reactive oxygen species to combat microbes invading the midgut [28]. The trypanosomes that escaped destruction had crossed the peritrophic matrix into the ectoperitrophic space, first observed on day 4 after infection (Fig. 6) [24].

**Fig. 7:**
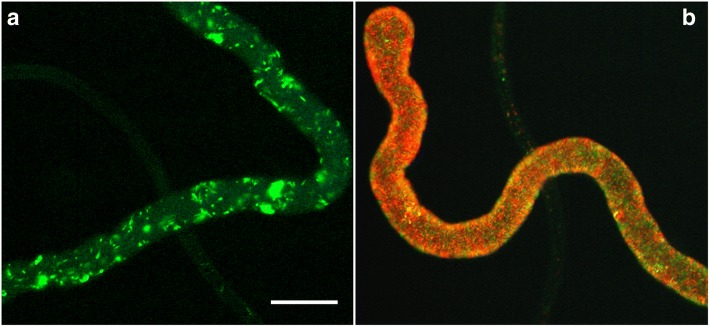
Fluorescent trypanosomes in salivary gland. Paired salivary glands from a single fly dissected 4 weeks after infection with red and green fluorescent trypanosomes. In panel **a** the salivary gland contains only green fluorescent trypanosomes, while in panel **b** the gland has a mixed infection of red and green fluorescent trypanosomes. *Scale-bar*: 500 μm. Copyright: Creative Commons Attribution 4.0 License (https://creativecommons.org/licenses/by/4.0/). Citation: Peacock et al. (2007). Dynamics of infection and competition between two strains of *Trypanosoma brucei brucei* in the tsetse fly observed using fluorescent markers. *Kinetoplastid Biology and Disease.* 2007;6:4 [29]

When flies were fed approximately equal numbers of red and green fluorescent trypanosomes, after dissection the overall midgut infection rate was about 55% and the majority were mixed infections, suggesting little competition between trypanosome strains [29]. However, the salivary gland infection rate was much lower (3.6%; 60/1663) and only 37% of these flies had a mixed infection in one or both glands (22/60), indicating a severe bottleneck in establishing a mature infection. Tellingly, in more than half of the flies (57%; 34/60), the infection in the paired salivary glands did not match (Fig. 7; Table 1), indicating that each gland had been colonised independently and probably only by a small founder population of trypanosomes, as less than a third of individual salivary glands contained both red and green fluorescent trypanosomes (29%; 35/120). Similar results were found in a study using sequence-tagged trypanosome clones [30]. It is not clear what causes this bottleneck but contributory factors may be: failure of the trypanosomes to differentiate into migratory forms, losses during migration, a hostile environment when trypanosomes reach the salivary glands, failure to differentiate and proliferate in the salivary glands.

**Table 1:** Independent colonisation of the salivary glands. Salivary glands infection profiles of 60 flies infected with red and green fluorescent *Trypanosoma brucei*. Data from [29]

Paired salivary glands from individual flies	No. of flies
Trypanosome population identical in both glands (both red, both green or both mixed infection)	26
Trypanosome population differs between glands (red + green, red + mixed infection, green + mixed infection)	12
Only one gland infected (red, green or mixed infection)	22
Total	60

## Conclusions

**Fig. 8:**
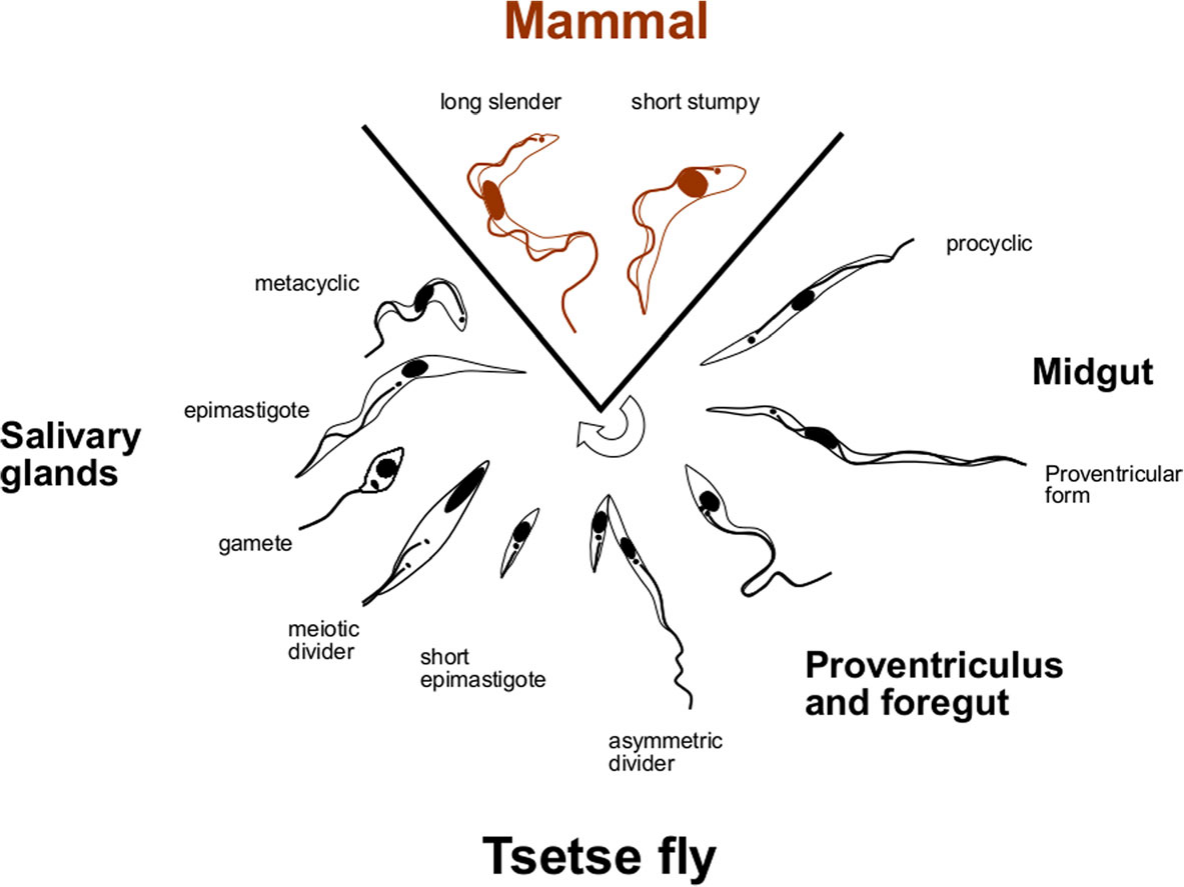
Life-cycle diagram. Diagram of the life-cycle of *Trypanosoma brucei*, including sexual stages

Fluorescent proteins have created new possibilities for investigating tsetse-trypanosome interactions and enabled real breakthroughs in our understanding of mating and the mechanism of sexual reproduction in trypanosomes. We can now confidently include stages involved in sexual reproduction to the original life-cycle diagram devised by Keith Vickerman [1], as well as the migratory stages from the proventriculus and foregut [31, 32] (Fig. 8).

## Abbreviations

GFP: Green fluorescent protein
RFP: Red fluorescent protein
YFP: Yellow fluorescent protein
